# Influence of library relocation and marketing: examining zip codes and health disparities to serve consumers in East Tennessee

**DOI:** 10.5195/jmla.2020.965

**Published:** 2020-10-01

**Authors:** David W. Petersen, Martha Earl, Cameron Watson, Kelsey Grabeel

**Affiliations:** 1 dpetersen@utmck.edu, Assistant Professor and Research and Learning Services Librarian, Preston Medical Library, University of Tennessee Graduate School of Medicine/University of Tennessee Medical Center, Knoxville, TN; 2 mearl@utmck.edu, Associate Professor and Director, Preston Medical Library, University of Tennessee Graduate School of Medicine/University of Tennessee Medical Center, Knoxville, TN; 3 cwatson1@utmck.edu, Library Associate III, Preston Medical Library, University of Tennessee Graduate School of Medicine/University of Tennessee Medical Center, Knoxville, TN; 4 kgrabeel@utmck.edu, Associate Professor and Assistant Director, Health Information Center, University of Tennessee Graduate School of Medicine/University of Tennessee Medical Center, Knoxville, TN

## Abstract

**Background:**

In 2014, the Preston Medical Library underwent a radical change, moving from an academic office building to the main floor of a regional medical center. While the library had previously served the public, health information requests have substantially increased in volume due to the new location. Researchers analyzed request data to see if the service's reach has expanded to counties that previously had not used the service, to see which counties have requested the most health information, and to ascertain whether more requests are from counties with higher poverty rates.

**Case Presentation:**

Each health information request is logged with the subject nature and patron contact information. Consumer health request data were downloaded from the library database. Names and other identifying data were removed. Request forms were sorted and reviewed by zip code and county, comparing number of requests as well as poverty levels. Tableau was utilized to create maps, visually showing patron concentrations and poverty levels.

**Conclusions:**

There were 3,141 health information requests from September 21, 2014, to May 31, 2019. The majority of requests were from local counties. Requests were also received from counties that had not been previously reached and counties with elevated poverty levels. Collecting data on patron interactions is not only critical for institutional reporting, but also for community outreach. Understanding that data require taking additional steps to filter the information, assess local demographics, and customize library services. Researchers anticipate being able to better tailor services to the community based on the results.

## BACKGROUND

Providing consumer health information to patients, families, and the public is a central mission for many hospital libraries. Libraries have taken several approaches toward fulfilling this mission, including featuring exhibits, displaying brochures, advertising services in the hospital, and conducting outreach events. Hospital libraries can quickly respond to patient and family inquiries about health conditions. Despite being an important resource, hospital libraries have frequently been forced to close or consolidate, in part due to a perceived lack of return on investment [1]. Budget restraints and lack of administrative buy-in can make it difficult for the remaining libraries to prosper [2]. However, the Preston Medical Library (PML), which serves both the University of Tennessee Graduate School of Medicine (UTGSM) and the University of Tennessee Medical Center (UTMC), has managed to expand services through a focus on the public. This case study examines how PML had success with services, data collection, and data visualization, as well as offers workable ideas for other hospital libraries to demonstrate value and worth to their organizations.

Historically, PML, funded primarily by the University of Tennessee, has provided services to clinicians, faculty, staff, residents, and students. In 1993, PML expanded its services by developing a Consumer and Patient Health Information Service (CAPHIS) to provide community members with requested health-related information from the library for free. Library staff would use reliable sources to search for health information and then mail the information. Once the request was completed, staff entered the search information into an internal database with the subject matter and patron contact information.

To learn more about the population using CAPHIS, researchers conducted a study in 2012 analyzing use of the service, residency of those people, and demographic data (e.g., poverty, disability) associated with the requestors' geographical locations [3]. Demographic data were obtained through the US Census Bureau. Tableau was then utilized to visually map the data. User data were examined to see where the library should focus outreach. The primary limitation was that the 2012 study data reflected the library's status before moving inside UTMC and restructuring to include the Health Information Center (HIC).

In 2014, PML expanded by opening the HIC, a patient- and family-focused library, and moving to the main floor of UTMC, which put the library directly at the center of the hospital and offered access to both staff and patients. This move coincided with an increased marketing campaign to highlight the HIC in physician waiting rooms as well as gain physician and staff support to recommend library services. The result was a substantial increase in patient and consumer health information requests as well as other requests. A 2016 study of the HIC at UTMC discussed the library's increase in statistics and patrons due to the location change but did not answer certain demographic questions [4].

Therefore, the authors analyzed the internal CAPHIS database to identify the locations from which patrons were requesting information, specifically focusing on rural counties or zip codes with high rates of poverty. We examined the zip codes and counties of patrons who used the service but did not previously use it in the 2012 study [3]. We also examined the data to determine where to conduct future outreach.

## CASE PRESENTATION

With the struggles and closures of many rural hospitals, regional city hospitals are increasingly serving rural populations [5]. UTMC has a 21-county service area that includes several rural counties with high poverty scores and low education levels. Of those 21 counties, 6 had a poverty level above 20%: Claiborne, Cocke, Hancock, McMinn, Morgan, and Scott. Fewer than 10% of the population in Hancock, Morgan, and Scott counties hold a bachelor's degree or higher [6].

To identify the counties of patrons who were using the health information service, we collected data from the CAPHIS database and obtained UTGSM Institutional Review Board (IRB) approval to perform an analysis. The data were split into two sets: PML at the former location in an adjacent UTGSM building (1997–September 2014) and PML/HIC inside the hospital (October 2014–May 2019). Requests had both county and zip code listed; however, some were missing data. For those that only had the zip code, a search was performed to determine which county the zip code belonged in. Some results had no data in the zip code category but had a county listed. These entries were pulled into the overall set for the county but were not assigned a zip code. We removed 383 results due to coming from either walk-in service or “Skylight” service (internal request service that patients can access from their rooms) that had no zip code or county information. These blank results had been assigned UTMC's 37920 zip code; therefore, the rest of the 37920 zip code entries were separated from the dataset to avoid skewing the study's results. Only entries that had “complete” data (more than a zip code and a generic name) were counted toward the total 37920 zip code count. Requests were sorted by county and zip code, comparing results before and after the move.

**Figure 1:**
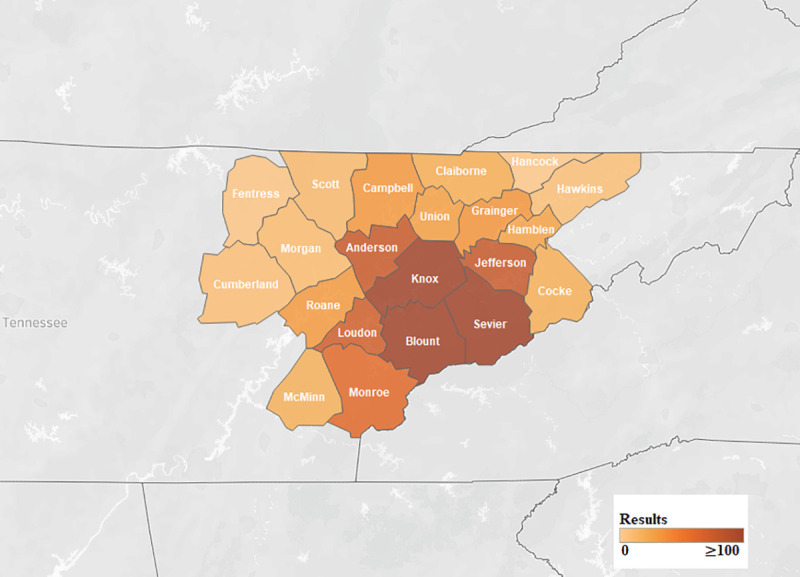
Requests by county in hospital's service area

**Figure 2:**
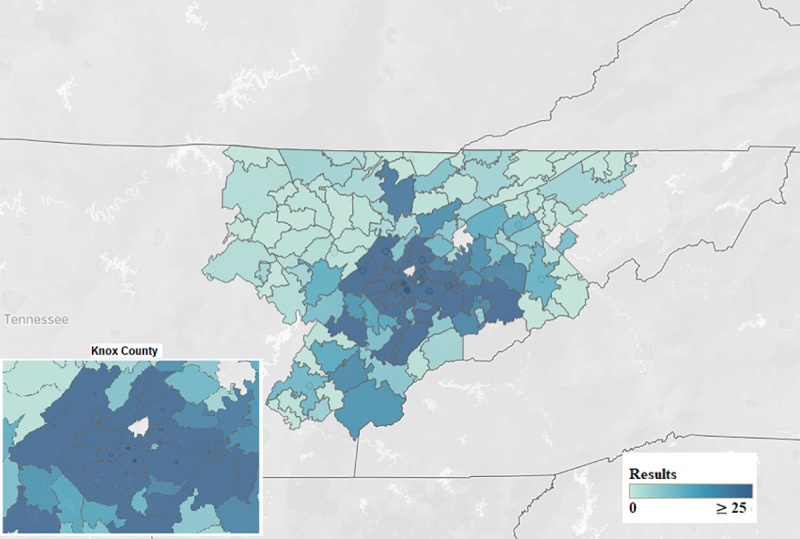
Requests by zip code in hospital's service area

We found that the number of consumer health requests increased after the move. From 1997 to September 2014, there were 3,801 requests (Figure 1). However, since the move inside the hospital and the opening of the HIC in September 2014, there were 3,141 requests by May 2019. At the UTGSM location, requests averaged 17.1 per month, whereas at the location inside the hospital, requests averaged 55.1 per month. Therefore, monthly requests for consumer health information increased by 208% since the move. The highest number of health information requests came from Knox and Blount counties, which was expected given that the hospital is located in Knox county (Figure 2).

When compared to the original 2012 study, the HIC now reaches 6 additional counties that were previously not using the service: Claiborne, Cocke, Fentress, Hawkins, Morgan, and Scott. Four of these counties have poverty levels greater than 20%. Hancock county is the only county in the hospital's service area that has not used the consumer health service, although it is the smallest county from a population standpoint, with only 6,819 residents according to the 2010 census. Hancock county also has the highest poverty level (28.4% of households).

**Figure 3:**
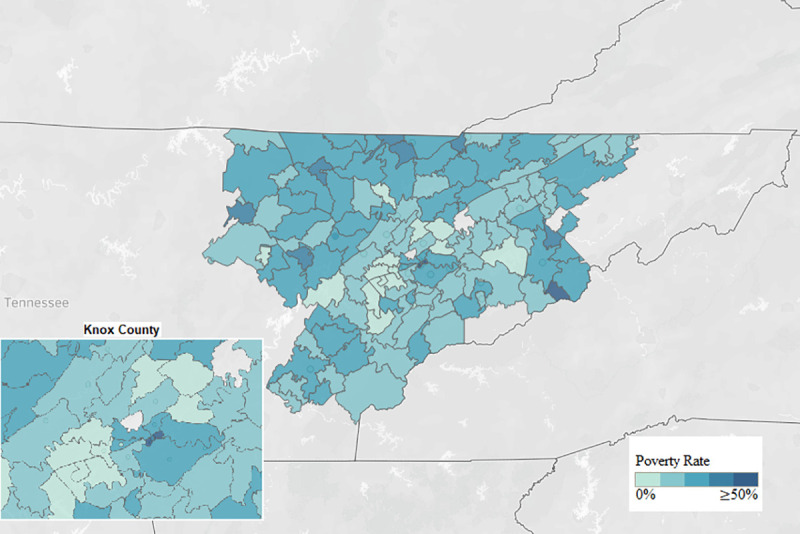
Poverty rates by zip code in hospital's service area

The HIC has received requests from more counties and zip codes, including many with a median income below the federal poverty line, since expanding inside the hospital and using new marketing strategies (Figure 3). Twelve Tennessee counties are ranked in the “Persistent Poverty County” listing, with greater than 20% of the county's population below the poverty level from 1990–2018 [7]. The HIC's information service has reached 5 of those counties. These data show that the HIC is actively reaching people in the hospital's focus area.

Finally, we found an increase in usage by patrons in Knox county. Patrons in the following three new zip codes in Knox county, from which we previously had received no health information requests, utilized the service since the opening: 37932, 37754, and 37806. Two other zip codes associated with very few requests in the 2012 study now had over twenty-five requests each: 37931 and 37909.

## DISCUSSION

The HIC's consumer health service now reaches twenty out of the twenty-one counties served, showing how the new location inside the hospital helped make an impact on providing more health information to patients, family members, and the community. In providing easy-to-read health information to the community, this free service is beneficial, particularly given that many of the counties served have a high poverty level percentage. By reaching these rural counties with high poverty levels, the HIC is working toward decreasing health disparities in the community [8, 9].

Throughout the last decade, hospital libraries have primarily focused on staying open and relevant to their institutions' missions. With administrative cost cutting, hospital libraries are frequently targeted to save money [2]. Taylor and Stephenson discussed a survey that federal medical center libraries utilized to prove the libraries' monetary value to the institutions, the results of which indicated that library-provided information assisted in decreasing length of stay and avoided increased costs [10]. Also, a 2009 study analyzed the literature and found that “hospital librarians and their services provide an excellent return on investment” [1]. The same year, however, a medical librarian stated, “There are no clear predictors of success or of failure [for hospital libraries].” Still, some hospital libraries have managed to thrive in this environment [11, 12]. Hospital libraries have focused on outreach, both to staff and the public, while also supporting “the mission of the hospital” [13].

Since the HIC supports the mission of UTMC by providing health information to patients and consumers in almost all of the primary zip codes that UTMC serves, the HIC potentially saves the hospital money, as Taylor and Stephenson discussed [10]. The new location in the heart of the hospital has increased the number of walk-in health requests. In addition, hospital marketing strategies related to the new location, fuller involvement of the HIC services in a more accessible location, and focused health literacy outreach to nurses on the floors has also helped to highlight the library's presence.

Within the first three months of relocation, the library began placing promotional materials, including brochures and table tents, around UTMC. The locations included high traffic areas in nearly every building on the UTMC campus, such as patient registration and waiting rooms. Physicians allowed the library to put brochures and information in their waiting rooms. Additionally, the library went on a local news station to promote the HIC and its services for the first two years after opening. After two years, the library began organizing pop-ups in which the library set up a booth in waiting rooms to advertise services and participated in cooperative events, such as local farmers' markets. Since the HIC opened, librarians have continuously provided outreach to nurses on the health information service by attending huddles and unit council meetings yearly.

Further outreach to Hancock county needs to be done, as that is currently the only county from which the HIC has not received health information requests. Currently, library staff attend remote area medical outreach programs to reach more of the rural community in the regional area. There are also plans for more outreach within the hospital to encourage staff and providers to tell patients about the health information service.

This article analyzes one particular library's experience. However, this case can speak more broadly to hospital libraries, even if most hospital libraries do not have a prime location in the hospital, extensive back data, or funding for a dedicated consumer health division. While each library's situation is unique, libraries can gain physician and staff support to put information about their services in waiting rooms and have staff recommend the library to the public when information is needed. Other ideas include signage throughout the hospital, where appropriate. By noting key facts that hospital administrators are interested in learning, librarians can collect and analyze data related to those points. Multiple tools, including Tableau, enable librarians to create compelling data visualizations for hospital administrators. Therefore, librarians can support the mission and vision of their hospitals while having the data to prove their value. PML and HIC have used the collected data to show hospital administration the impact that the health information service can have on the service area.

## Data Availability

Data associated with this article are available at the University of Tennessee Health Science Center Digital Commons: https://dc.uthsc.edu/gsmk_facpubs/13/.
